# Design and implementation of an intensive panel survey with refugees and other migrants in need of protection in Costa Rica

**DOI:** 10.1371/journal.pone.0301135

**Published:** 2024-03-28

**Authors:** Abigail Weitzman, Matthew Blanton, Sophie M. Morse, Gilbert Brenes Camacho, María José Chaves Groh

**Affiliations:** 1 Department of Sociology, University of Texas at Austin, Austin, Texas, United States of America; 2 Population Research Center, University of Texas at Austin, Austin, Texas, United States of America; 3 Philip R. Lee Institute for Health Policy Studies, University of California San Francisco, San Francisco, California, United States of America; 4 Centro Centroamericano de Población, Universidad de Costa Rica, San José, Costa Rica; 5 Escuela de Estadística, Universidad de Costa Rica, San José, Costa Rica; 6 Escuela de Estudios Generales, Universidad de Costa Rica, San José, Costa Rica; 7 Centro de Investigación en Estudios de la Mujer, Universidad de Costa Rica, San José, Costa Rica; Caleb University, NIGERIA

## Abstract

Over the last decade, the global population of refugees and other migrants in need of international protection (MNP) has more than doubled. Despite their rapid growth, panel data collection among MNP remains rare, leaving scholars with few data sources to draw on to understand dynamic changes in their social, economic, legal, or health circumstances. With that paucity in mind, we developed and piloted the *Encuesta de Refugiados*: *Experiencias Sociales y Salud* (ERESS), a weekly panel survey conducted with MNP living in Costa Rica. To our knowledge, this panel constitutes one of the first weekly surveys with MNP anywhere in the world. Here, we describe the overall study design, sample recruitment and retention, and key descriptive findings. We show that retaining demographically and socioeconomically diverse MNP in intensive panel surveys is possible and that doing so reveals valuable insights into dynamic changes in their incorporation, family dynamics, and health and wellbeing. By offering a summary of our field experiences and central methodological findings, we highlight the potential benefits and challenges of collecting intensive panel data with MNP, as scholars increasingly seek to understand their pre- and post-migration trajectories and relationships between the two.

## Introduction

The vast majority of scholarship on migrants and migrant health centers around individuals who have left their country of origin to pursue economic and/or social gains (for reviews see [1–3]). “Migrants in need of international protection” (MNP)—refugees and internationally displaced populations in “refugee-like situations” [4] who migrate abroad to evade imminent threats to their survival—remain notably absent from the migrant populations most often studied. Yet, as a global population, they have increased more than threefold in the last decade, from 16.1 million in 2012 to 50.5 million in 2022 [5]. The swift global growth of MNP populations underscores the need for new theoretical frameworks and data sources to foster greater understanding of their migration dynamics, incorporation, family life, and health.

Presently, few panel data sources—with repeated observations from the same migrants—exist to facilitate such research. The longstanding dearth of comprehensive, repeat micro-data on MNP reflects the multiple barriers to recruiting and retaining these populations in scientific studies. With respect to the former, MNP are typically a “hidden” population. Although mainstream media portrayals convey MNP as residing in refugee camps, the reality is quite different—only an estimated 22% formally live in camps [6]. The vast majority thus reside among the general population in a given country. What is more, not all MNP apply for asylum or register with the United Nations once abroad. In most receiving contexts, there is therefore no reliable sampling frame for MNP. Further complicating matters is the fact that many MNP confront poverty once abroad [7–9]. Consequently, recruiting and maintaining contact with them by phone or Internet can prove difficult. Migrants, especially women, experience high risk of violence during their migration trajectories [10,11]. Depending on cultural norms and violence exposures before, during, or after migration, MNP may also be suspicious or mistrusting of researchers in ways that further impede their recruitment and retention in panel surveys [12–14].

**Fig 1:**
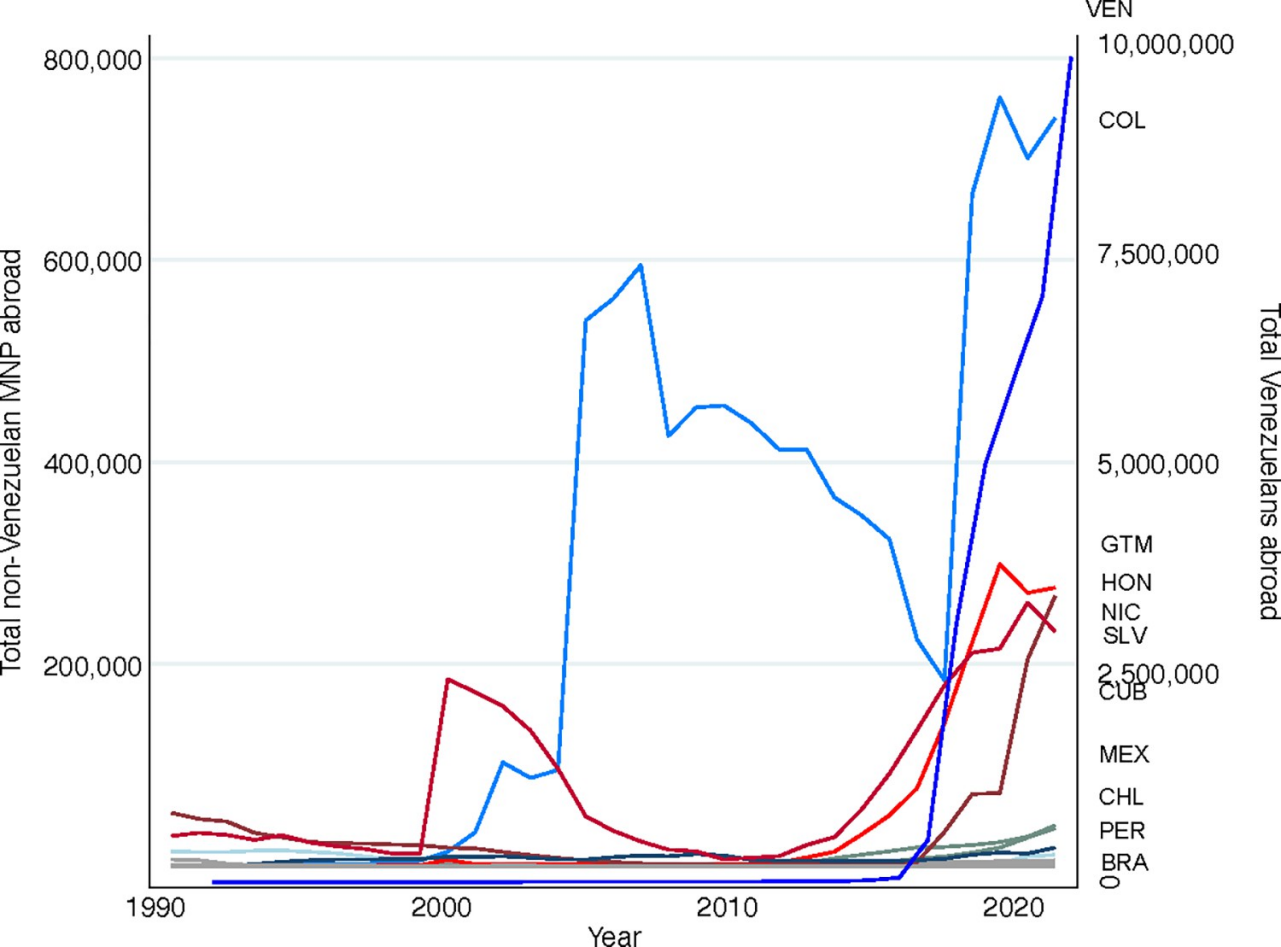
Latin American migrants in need of protection (MNP) abroad. Estimates calculated from data from the UNHCR Refugee Data Finder (https://www.unhcr.org/refugee-statistics/download/?url=2bxU2f).

With both the need for longitudinal micro-data on MNP and the challenges to cultivating such data sources in mind, we developed and piloted a weekly panel survey with MNP living in Costa Rica. To our knowledge, our pilot represents one of the first intensive panel surveys collected with MNP anywhere in the world. Our focus on Costa Rica is motivated by the incredible growth of Latin American MNP populations in recent decades and the fact that an estimated 76% of MNP resettle in lower- or middle-income countries in the global South [15]. As Fig 1 illustrates, nearly 10 million Venezuelan MNP have left Venezuela in the last seven years. Colombian, Cuban, Guatemalan, Honduran, Nicaraguan, Mexican, and Salvadoran MNP, too, are migrating abroad in record numbers (Fig 1). Currently, we know little about how their migration unfolds or their incorporation abroad; and we know even less about how these processes are inter-connected in ways that affect their short- and longer-term socioeconomic, family, and health trajectories.

**Fig 2:**
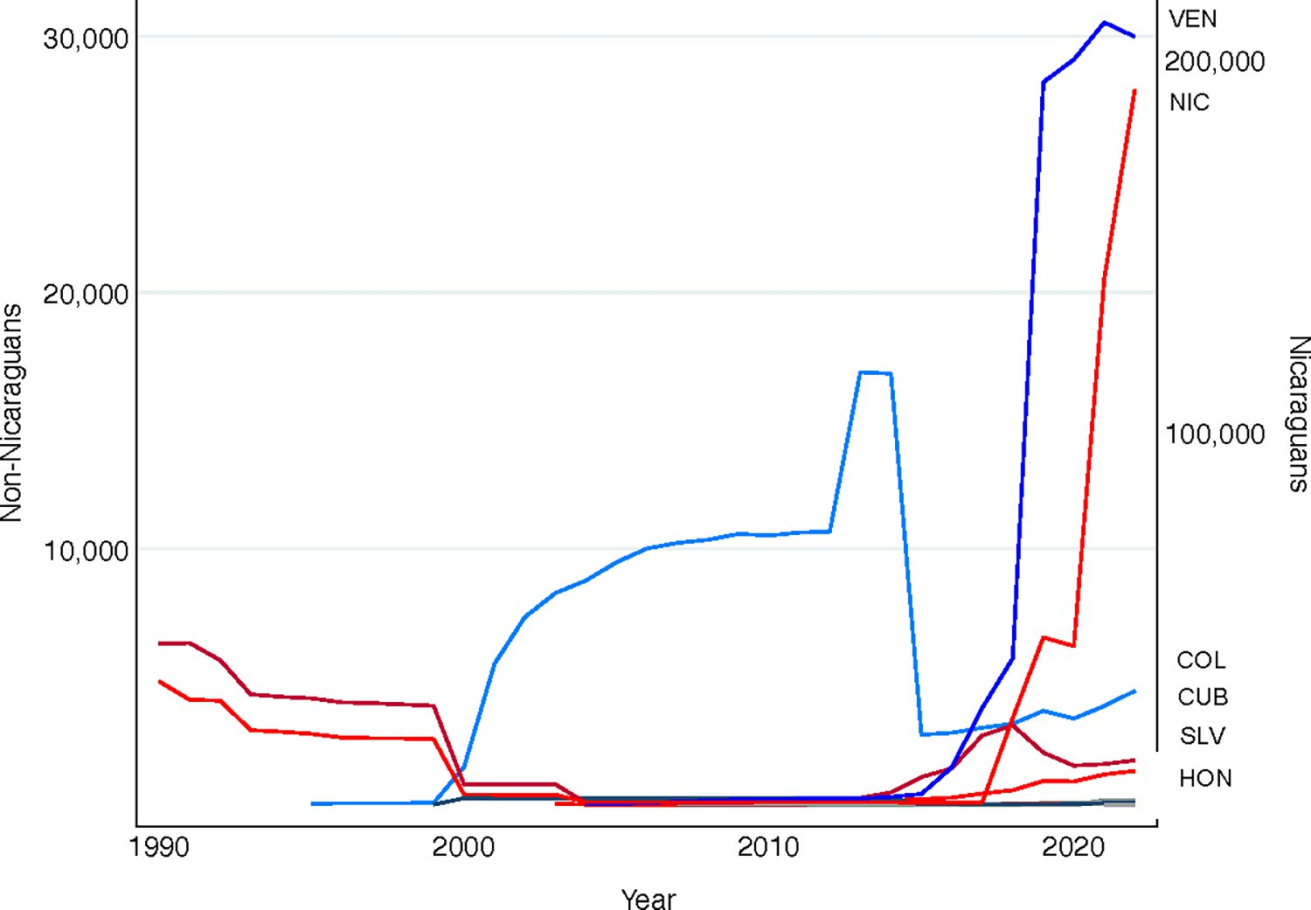
Latin American migrants in need of protection (MNP) in Costa Rica. Estimates calculated from data from the UNHCR Refugee Data Finder (https://www.unhcr.org/refugee-statistics/download/?url=2bxU2f).

Costa Rica offers a strategic location to investigate these issues because it is a middle-income country and a new migration destination for hundreds of thousands of MNP [16,17]. Both the number of Nicaraguan and Venezuelan MNP in Costa Rica have skyrocketed in the last five years, alongside steady increases in Colombian, Cuban, Salvadoran, and Honduran MNP populations (Fig 2). Venezuelans and Colombians regularly traverse the Darien Gap, one of the world’s most treacherous migration routes, in their desire to seek refuge in Costa Rica [18,19]. Migrants originating from these countries represent a diversity of sociodemographic profiles, cultural and political experiences, and precipitating push factors. Most Venezuelan MNP migrate abroad because of food and medical shortages, economic deprivation, political conflict, and other disturbances to public order [20,21]. Meanwhile, MNP from northern Central American countries like El Salvador and Honduras typically report migrating because of gang or gender-based violence [22,23]. Nicaraguan MNP, too, sometimes migrate because of gender-based violence but the vast majority report migrating because of political repression and violent political conflict [20,24]. These precipitating conditions in and of themselves affect a person’s legal, social, and economic incorporation (or not) in a given destination [20,25]. Yet, incorporation and corresponding family and health trajectories also vary by age, gender, racial, and class backgrounds [26,27], begging the need for comprehensive longitudinal data that can capture these dynamic trajectories and heterogeneity therein.

In the pages that follow, we detail the types of dynamism that extant scholarship leads us to expect among MNP and briefly describe the handful of micro-data sources that exist to investigate this dynamism, albeit at wide intervals of time (typically six months to a year apart). Following, we describe our study’s innovative approach to capturing dynamism in the socioeconomic circumstances, incorporation, family dynamics, and health and wellbeing of MNP. We then show that with situationally attuned protocols, MNP from diverse national and demographic backgrounds can successfully be retained in intensive panel studies at nearly comparable levels. Drawing on socioeconomic conditions as an illustrative case in point, we further highlight the types of insights that scholars can glean from frequent follow-up surveys. To conclude, we discuss the potential for expanding intensive longitudinal surveys among MNP and highlight the valuable theoretical and empirical implications that would stem from such efforts.

### Incorporation dynamism in the lives of migrants in need of protection

Migrants in need of protection (MNP) typically contend with high levels of adversity and uncertainty, often for years. With respect to legal incorporation, refugee and asylum applications can take years to process [28]. In the Costa Rican case, for example, recently arrived asylum seekers currently wait up to ten years for an asylum interview [29]. In the meantime, they are assigned a temporary status known as *solicitante* that has to be renewed in-person every two years [30]. In other contexts, select groups of MNP are granted a temporary protected status that is renewable after several years if circumstances in their country of origin are still deemed unsafe. Such is the case for select Salvadorans in the United States [31,32] and select Venezuelans in Colombia [33]. In Costa Rica, for certain MNPs from Nicaragua, Venezuela and Cuba, a two-year temporary protection program has been put in place allowing asylum seekers an option for impermanent regularization through a different pathway [34]. Whether awaiting an asylum interview or granted TPS, MNP find themselves in legal limbo, not knowing how long they will be allowed to remain in their current destination or whether they will eventually be granted a permanent immigration status. On the other hand, some never apply for refugee, asylum, or protected status and instead reside in a given destination with irregular status. These individuals, too, live in limbo in that their irregular status places them outside formal labor markets and healthcare systems [35,36] and they face the risk of sanctions or deportation if discovered by legal authorities [37,38].

Economically, MNP often also face turbulence. Depending on their immigration status and destination setting, they may not be allowed to enter the formal labor market or transfer their occupational credentials from their country of origin [27,39,40]. Even if granted a work permit or in-country credentials, MNP often report high levels of labor market discrimination across contexts of reception [41]. This discrimination can depress their wages [42] and make it hard for them to obtain or maintain employment [41,43]. Income insecurity, in turn, can result in bouts of hunger or homelessness [44–46] and likewise can exacerbate residential instability in the form of evictions [44] and/or household compositional changes that accompany a churning of household members [47].

Moreover, MNPs’ economic insecurity is often compounded by the fact that their migration was somewhat abrupt and unforeseen [48]. This means that many do not have extensive social networks to draw on abroad [49,50]. Even when they do, their prolonged hardships and stressors can strain their relationships with kin who live in the same destination context [49,51]. Institutional supports, such as NGOs and public ministries, too, are often stretched too thin to support MNP with regularity [52]. This is especially the case in countries like Costa Rica, where the recent growth of MNP is unprecedented.

Legal, economic, and social incorporation, in turn, have implications for MNPs’ evolving family dynamics [26,27]. Multiple qualitative studies, for instance, document that displaced families’ stress adjustment processes ebb and flow over the course of years [39,44], alongside changes in parents’ and children’s legal and socioeconomic circumstances [51]. These stress adjustment processes unfold between multiple family members who have divergent experiences *in the same place* abroad, but MNPs’ transnational family structure often also changes with time. For example, some family members depart origin sooner than others or become separated during the migration journey [48,53]. Once abroad, migrants’ legal status further shapes their possibilities for family reunification [54]. Changes in legal immigration status can thus prompt changes in family structure [54]. When this happens, parent-child and spousal dynamics often differ from their pre-migration dynamics because of the time spent apart and each individuals’ feelings and experiences during separation [54]. Interpersonal changes, combined with legal and socioeconomic stressors, may exacerbate family conflict. One cross-sectional study with sub-Saharan migrants in France, for instance, finds that having irregular documentation status is associated with higher odds of forced sex from intimate partners [55]. Likewise, a cross-sectional survey in Ecuador finds that discrimination and other psychosocial stressors are associated with elevated odds of IPV among Latin American refugees [56]. Both studies suggest that family conflict and violence may be sensitive to MNPs’ changing stressors, though panel data are needed to fully assess this possibility. At least one cross-sectional study finds that levels of family conflict among Rohingya and Afghan refugees increase with months in-destination (Malaysia) [57].

MNPs’ incorporation and family dynamics also have implications for changes in their health and wellbeing, and vice versa. For example, migrants’ stress levels, depression, and optimism for the future may vary as a function of their evolving legal or employment status [28], perceived discrimination [58], or residential instability [59]. Changes in these circumstances should also produce changes in their physical health via mental health somatization [58]; sanitation and residential conditions [60]; and/or sustained nutritional changes [46].

In short, qualitative and cross-sectional studies make clear that many MNP contend with perpetual adversities and uncertainties for multiple years after arriving to a destination. These challenges have both short and potentially longer-term ramifications for their family life, health and wellbeing.

### Current panel data sources on MNP

Understanding how these dynamic changes unfold over time necessitates panel data that are able to observe the same MNP repeatedly. Presently, only a handful of such data sources exist. These include the *Building a New Life in Australia Study*, which surveyed permanent humanitarian visa holders in Australia annually for five years [61]; the *Somali Youth Longitudinal Study*, which surveyed foreign-born young-adult Somalis in the U.S. four times over seven years [62]; the *Indochinese Health and Adaptation Research Project*, which surveyed southeast Asian refugees in San Diego twice in three years [63]; and the *Cox’s Bazar Panel Survey*, which included four waves of semi-annual surveys among Rohingya refugees and receiving community members in Bangladesh [64]. In addition, they include the *Syrian Refugee Life Study*, which surveyed registered Syrian refugee households in Jordan up to three times, three months apart [65] and the *UNHCR Socioeconomic Impact of Covid-19 on Forcibly Displaced Populations* surveys, which surveyed MNP who were utilizing UNHCR services between two and five times in several different countries of reception (for more information, see: https://microdata.unhcr.org); among several other surveys.

These data sources offer critical opportunities to understand how the experience of MNP incorporation unfolds. At the same time, each was collected at wide temporal intervals: With the exception of the *Syrian Refugee Life Study*, these panel surveys re-assessed participants at intervals of six months or longer. Thus, while they illuminate MNPs’ trajectories for upwards of several years, they are largely unable to reveal how changes in one facet of MNPs’ lives, such as job loss, can quickly have cascading effects on other facets of their lives, such as residential instability. Short temporal spacing is needed to develop more fine-grained understandings of the *temporal ordering* and *dynamics* by which these changes unfold. For instance, it may be the case that job loss leads to residential change, but the reverse may also be true—a possibility that can only be accurately determined when the two changes occurred in between different waves of survey reporting. Likewise, frequent assessments are needed to gauge the typical *amount of time* between job loss and residential change and to identify intervening pathways such as corresponding changes in household composition, childcare lapses, household expenditure, and mental health. Answering questions relating to temporality and identifying *immediate* intervening pathways is imperative to understanding MNPs’ needs, behaviors, and overall demographic patterns.

Notably, current panel surveys of MNP largely focus on refugees, asylees, or other humanitarian visa holders: All but the UNHCR samples are restricted based on immigration status. These surveys thus cannot enable understandings of how legal immigration status shapes MNPs’ trajectories over time nor can they reveal how different types of precipitating threats (e.g. political persecution, gender-based violence, gang violence, etc.) place MNP on divergent legal trajectories. Relatedly, most available longitudinal micro-data are collected among MNP in high-income receiving contexts or in refugee camps, despite the fact that these settings host a small minority of MNP. Notable exceptions include several UNHCR surveys that are collected outside of camps and the *Syrian Refugee Life Study* and *Cox’s Bazar Panel Survey*, both of which include a combination of MNP who do and don’t live in camps. These latter two surveys are thus uniquely well positioned to reveal residentially-based differences in MNPs’ incorporation, family, and health trajectories. Besides select *UNHCR Socioeconomic Impact of Covid-19 on Forcibly Displaced Populations* surveys, which collected two rounds of data on Covid-19 related experiences and household income and expenditure in Mexico and Costa Rica, to our knowledge, no panel micro-data exist on MNP originating from or residing in Latin America, even though these sub-populations demonstrate immense growth (Fig 1).

## Methods

### Study context and design

Costa Rica is a small Central American country with approximately 5.1 million residents [66]. Today, an estimated 4% of Costa Rican residents are MNP [29]. These individuals reflect a combination of north- and south-bound migration (Fig 2), owing in part to Costa Rica’s geographic location, which is nestled between South America and northern Central America. Though its migration politics are beginning to change [52], Costa Rica has historically been considered a hospitable destination for MNP [16]. For instance, Costa Rica possesses only one small detention center and rarely detains migrants, including when they have irregular status [67]. Likewise, rather than being deported, applicants who are denied asylum can appeal their denial and have historically been encouraged to find an alternative means of obtaining legal immigration status [68]. Beyond these policies, Costa Rica’s strong public education and healthcare systems, political stability, and relatively egalitarian gender norms also contribute to its appeal [16].

Our study design was informed by the ethno-survey methodology, in which “ethnographic and survey methods inform one another throughout the study” (p.1505) [69]. Data collection thusly began with a qualitative scope study designed to uncover methodological challenges to retaining MNP in longitudinal surveys; and to assess, from MNPs’ vantage point, what were the major themes characterizing their migration, incorporation, family dynamics, and health and wellbeing. Following the scope study, we conducted a pre-pilot survey assessing the feasibility of virtual recruitment and survey completion. We then implemented our panel survey, including a baseline survey and twelve weeks of weekly follow-ups. Each phase of research informed the subsequent phase.

Our research team consisted of a project Principal Investigator from the United States and co-Investigator from Costa Rica, as well as four research assistants from these same countries. Before commencing with data collection, we consulted with social workers and trauma counselors who specifically work with Latin American MNP populations in the United States and in Costa Rica and continued to consult with these individuals throughout the research process to ensure that our protocols were culturally and situationally appropriate for individuals from a range of Latin American countries who had experienced varying degrees of trauma. To further minimize potential harm, for all data collection, we partnered with and primarily recruited through an NGO that has more than 25 years of experience serving the MNP population in Costa Rica, known as Fundación Mujer. (The organization’s name is a relic of its history. It does not exclusively provide services to women despite its name.) Because Fundación Mujer conducts intake with their clientele, and has substantial experience working with MNP, they were able to specifically refer individuals who they believed were emotionally prepared to talk about their experiences with the research team. We also allowed for snowballing, but only after someone had participated in the survey and was able to share information about what the experience was like with potential recruits.

### Scope study

The scope study included informal interviews with MNP service providers and focus groups and in-depth interviews with women MNP. First, in 2018 and 2019, we conducted informal background interviews with service providers from a range of local and international NGOs and public offices. These interviews offered contextual information about the asylum process and eligibility, MNP’s rights, and perceptions of their geographic residence, economic conditions, living arrangements, interpersonal dynamics, and interactions with institutions. Through these informal interviews, for instance, service providers repeatedly emphasized that MNP change phone numbers with regularity in a way that makes it difficult to recontact them. They also speculated that some are suspicious of their compatriots and offered examples to backup this perception. We took this information into account when designing the focus group protocols and questionnaire.

On November 28 and 29, 2019, we conducted four focus groups with a total of 44 women MNP from Colombia, Cuba, El Salvador, Honduras, Nicaragua, and Venezuela. Focus groups were recruited from and conducted at Fundación Mujer. Participants gave oral informed consent and were remunerated $20 cash for their participation. During these focus groups, we asked participants how they stayed in touch with their loved ones in origin; the best ways we as researchers could stay in touch with them; their ownership or co-ownership of cell-phones; and how they accessed the Internet. We also asked how easy or difficult it would be for them to complete an online survey every week and why; and whether they knew anyone who would be hard to recruit and why. Additionally, we asked them to describe how their family life and health had changed since arrival.

Methodologically, we gleaned four important takeaways from these focus groups. First, MNP typically own or share a cellphone with someone in their household, which should enable them to complete phone surveys. Second, in urban areas of Costa Rica, free Wifi is available in many public spaces, including public parks and shopping malls. Many MNP can thus access the Internet with regularity. Third, because many have difficulty finding formal employment, MNPs’ daily and weekly schedules are hard to predict. In light of this unpredictability, participants encouraged us to offer online surveys and to give participants up to a week to complete them. Fourth, establishing interpersonal rapport with MNP is paramount to earning their trust and participation. Each of these findings informed our subsequent survey design.

After the focus groups, from January 31 to March 13, 2020, we conducted in-depth interviews with 34 women MNP from Cuba, El Salvador, Nicaragua, Venezuela, and a less common South American country of origin (intentionally withheld to protect the participant’s identity). Interviews were also recruited from and conducted at Fundación Mujer; orally granted informed consent; and received $20 cash for participating. Through these interviews, we expounded upon our substantive focus group findings (on MNPs’ economic, family, and health conditions) to uncover the major themes characterizing their daily life. To better connect their pre- and post-migration experiences, we structured these in-depth interviews like life histories with an emphasis on family dynamics and interpersonal relationships at different points in time, starting early in the life course and continuing to the present day. The themes arising from these interviews, in conjunction with extant literature, also informed the content of our survey questionnaires. For instance, in both focus groups and in-depth interviews, the most commonly articulated ongoing stressors were family separation, downward economic mobility, cost of living, and housing. We therefore included questions about each of these topics in the baseline and follow-up surveys. Likewise, when asked about their health, focus group and in-depth interview participants often referenced one or more of a common set of symptoms (digestive problems, lack of appetite, headaches, difficulty sleeping) and/or preexisting conditions. Correspondingly, the panel survey’s baseline questionnaire included a question about preexisting conditions and the baseline and follow-up questionnaire both included questions about these specific symptoms as well as symptoms of Covid-19.

### Pre-pilot phase

Based on our focus groups and in-depth interviews, our original plan was to conduct face-to-face baseline surveys followed by online follow-up surveys. The onset of the Covid-19 pandemic, however, upended these plans, which presented a major obstacle given the importance of rapport conveyed by our focus groups. We therefore conducted a pre-pilot survey to assess the feasibility of virtual survey recruitment and completion. As a first attempt, we asked Fundación Mujer to send a brief description of the survey and a survey link to 300 clients from diverse demographic backgrounds in March 2021. This strategy did not yield a single response even though every person who was contacted had at some point utilized Fundación Mujer’s services. So, as a second attempt, in May 2021, we accompanied Fundación Mujer during their regularly scheduled online programming, where they created space for us to offer brief information about the survey and answer questions about it. This strategy was far more successful and yielded a total sample of 148 pre-pilot survey participants who we recruited through Zoom and subsequent snowballing.

Pre-pilot survey participants completed surveys from May 25 to June 19, 2021; were given an online informed consent form and clicked a box consenting to the survey before participating; and received a $5 phone credit after completing the survey online if they opted to provide a phone number. The pre-pilot survey, which we called *Refugiado en Costa Rica Durante Covid-19* (ReCoRD Covid), took approximately 15 minutes to complete online and included questions about participants’ demographic characteristics, journey to Costa Rica, migration push factors, social network, physical health, current socioeconomic circumstances, immigration status, and experiences with Covid-19. Responses to the pre-pilot survey informed the development of the subsequent baseline questionnaire that was administered as part of the panel survey (described below). Online responses also bolstered our confidence that participants in a panel survey could successfully access the Internet and complete follow-up surveys online.

### Panel survey

In October 2021, we began recruiting participants for our panel survey, known as the *Encuesta de Refugiados*: *Experiencias Sociales y Salud* (ERESS) (Survey of Refugees: Social Experiences and Health). We included “refugees” in the survey name because, in Costa Rica, this word is the most common colloquial term used to refer to MNP. To be eligible, participants had to be ≥18 years-old, born outside of Costa Rica, and living in Costa Rica for at least six months. Because there is no definitive way to distinguish MNP from non-MNP migrants, we did not impose additional participation restrictions but instead continued to recruit from Fundación Mujer, who primarily serves MNP clientele. To ensure that *all* MNP felt welcome to participate—not just those who had applied for asylum—study team members followed a recruitment script that clarified that ERESS was intended to “understand how the experience of being displaced affects health” and that participation was open to “migrants and refugees…who were born outside of Costa Rica.”

**Fig 3:**
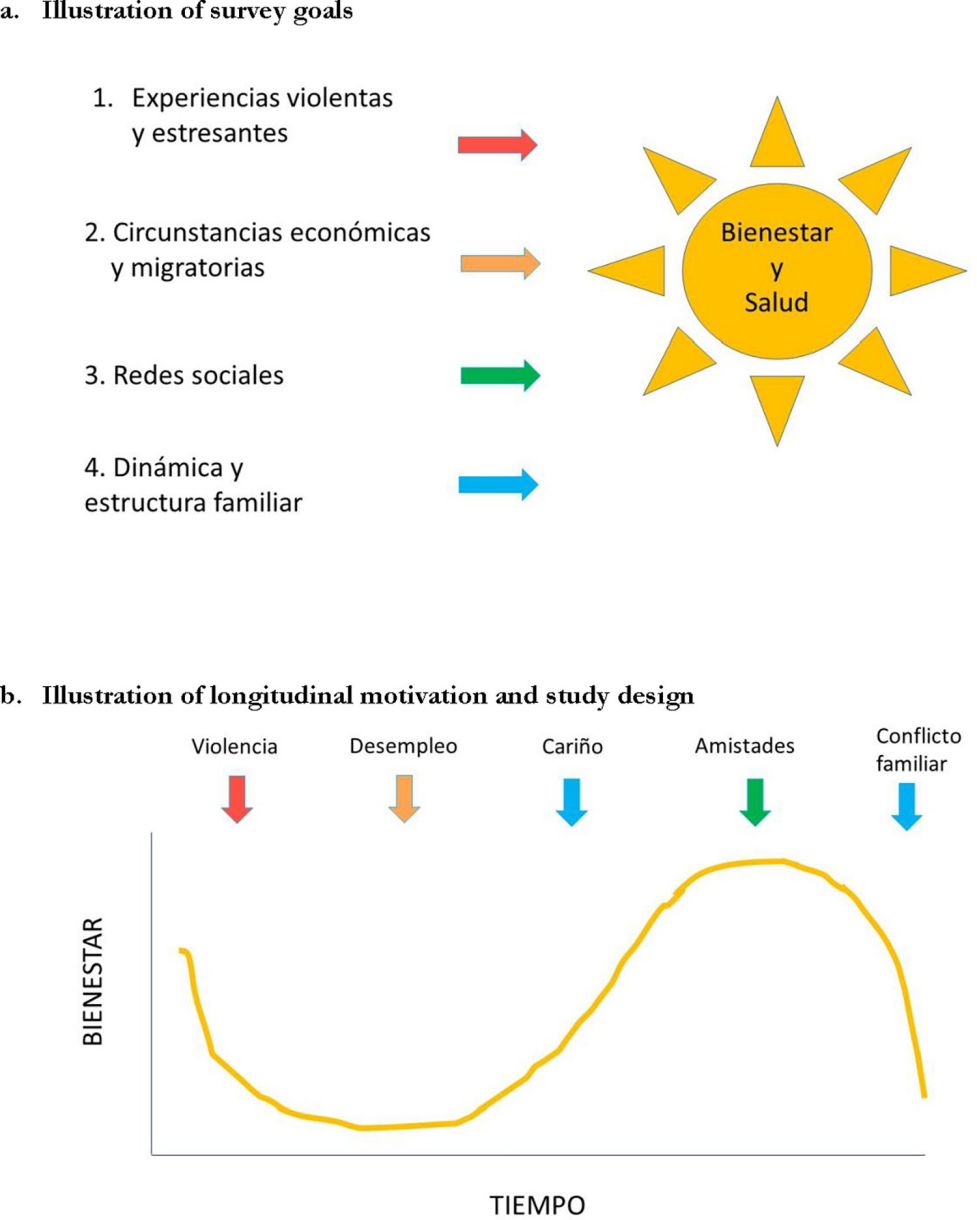
Infographics sent to ERESS participants.

A total of 260 participants were enrolled on a rolling basis, beginning October 14, 2021 and ending on April 2, 2022. Approximately the first third of these participants were recruited by recontacting pre-pilot survey participants who had provided a phone number. The remainder were recruited through online Zoom workshops with Fundación Mujer. Once a person expressed interest in participating, we began a process of rapport building and information sharing that typically lasted one to two weeks. Over WhatsApp (an encrypted, web-based text messaging application), two team members sent potential participants infographics about the survey and the purpose of follow-ups (Fig 3). They also outlined the survey process, answered questions, and sent participants informed consent documents to review. Throughout the entirety of ERESS, both team members remained “on call” to respond to any participant queries as they arose. Once a person agreed to participate, these same team members coordinated a time that was convenient for that person to complete the baseline survey by phone. Baseline surveys were scheduled day or night, seven days a week. Participants orally granted informed consent and received a $5 phone credit for taking the survey.

At the start of baseline, participants were reminded that their participation was anonymous and were given a randomized four-digit pin number to enter during the follow-up surveys. We then implemented the baseline questionnaire taking an ethno-survey approach [69]. That is, we asked survey questions as if they were open-ended, allowing the phone survey to feel more intimate and conversational. Most of the time, participants provided answers that fit within a given set of survey response options. When they did not, however, we reviewed the response options with them and asked them to choose one (or multiple). All participants completed the baseline survey and only a handful skipped any questions at all.

Baseline surveys typically lasted 35 minutes, though in rare cases, their conversational nature led some to last over an hour. Through these baseline surveys, we collected information on demographic background; household structure and transnational family ties; year of arrival; primary and secondary motivations for migrating; future migration plans; visa status; socioeconomic circumstances; social support, discrimination, and service utilization in Costa Rica; mental and physical health; local and transnational family dynamics; adverse childhood events and similar adverse adulthood events; and violence exposures in origin. Whenever possible, the specific wording of questions and responses was based on preexisting questionnaires with established validity among Latin American populations, such as the Demographic and Health Surveys (DHS), Latin American Public Opinion Project (LAPOP), Violence Against Children Survey (VACS), and censuses from the countries where most MNP in Costa Rica originate from.

Once enrolled, participants received a link to an online weekly survey every Friday for twelve consecutive weeks. To enter the survey, they inputted their four-digit pin number, which enabled us to track the same participants’ responses over time. If a participant had not completed the survey within four days of being sent the link, we sent them a WhatsApp reminder. Occasionally, participants forgot or lost their pin number and reached out to the study team to re-request it. Likewise, several participants lost or damaged their phones during the survey and reached out to us to inform us of their new number once they had one. At the start of each weekly survey, participants were asked to reread the informed consent document, which was presented to them online, and to click a box granting informed consent. They then answered a series of survey questions that repeatedly assessed their household composition; migration plans; visa status; socioeconomic circumstances; social support and discrimination; mental and physical health; and local and transnational family dynamics. Each week, we further included one of four rotating sets of questions about participants’ fertility desires; trust of others in Costa Rica; perceived resilience; and sense of belonging and nostalgia. These online surveys took roughly 10 minutes to complete and participants received a $5 phone credit each week that they took the online survey.

### Ethics statement

The research protocol was approved by the Institutional Review Board at The University of Texas at Austin (IRB #2019040084) and the University of Costa Rica (IRB #CEC-446-2019).

## Results

### ERESS sample

Our sample consists of 260 migrants. Although we cannot be entirely sure that all participants were MNP, >97% had either applied for asylum; reported that direct threats, general danger and insecurity, or political turmoil in their country was a key reason they migrated; and/or migrated from a country with a large humanitarian crisis in the year that they migrated. We thus feel confident that our sample primarily consists of migrants whose circumstances meet the general definition of MNP. Table 1 provides a descriptive overview of the sample. The S1 Table reviews how each indicator was assessed and the S2 Table compares our sample to a convenience sample of UNHCR-registered MNP in Costa Rica who participated in a UNHCR survey around the same time as ERESS.

**Table 1:** Descriptive characterization of ERESS.

	Respondents (N = 260)		Follow-ups (N = 2,866)	
	Mean	SD	Mean	SD
Participated in ≥1 follow-up	.95			
…Participated through last follow-up	.74			
…Last week of follow-up (1–12)	10.82	2.71		
…Participated in all follow-up weeks	.38			
…Number of follow-up weeks missing	1.21	1.42		
*Demographic background*				
Age (18–70)	38.62	11.50		
Woman*	.66			
Educational attainment				
< = Primary	.18			
Secondary	.18			
Technical	.27			
> = University	.36			
Relationship status (ref: married)				
Married	.33			
Cohabiting	.22			
Divorced, separated, widowed	.11			
Single	.35			
Any children in Costa Rica	.50			
Number of children in Costa Rica (0–4)	.87	1.04		
Race				
White	.21			
Mestizo	.54			
Black, Mulato, or Afrodescendant	.14			
Indigenous	.04			
Other	.07			
Nationality				
Nicaraguan	.50			
Venezuelan	.33			
Salvadoran	.07			
Other	.10			
*Sociolegal incorporation*				
Migration status				
Awaiting asylum interview	.63			
Asylee	.14			
Pending asylum appeal	.05			
Other visa	.14			
Undocumented or denied asylum & appeal	.04			
Years in Costa Rica				
<1	.08			
1-<2	.08			
2-<3	.17			
3-<4	.37			
> = 4	.30			
*Socioeconomic incorporation in Costa Rica*				
Changed residences (by baseline/ last week)	.86		.24	
Homeless (by baseline/ last week)	.50		.20	
Went hungry last week	.49		.38	
Generated income last week	.79		.94	
Did something not proud of to survive last week	.24		.33	

*Note*: The category “women” includes three trans-women.

On the whole, participants represented a diversity of demographic backgrounds. For instance, they ranged in age from 18 to 70 years old, with an average age of 38. Two-thirds identified as a woman, including three trans-women. Just over a third graduated from university, while 18% graduated from secondary, 27% graduated from technical (vocational secondary), and 18% had not attended or had not completed secondary school. Just over half were married or cohabiting, while approximately a third were single and 11% were divorced, separated, or widowed. Fifty percent of participants had children <18 years-old with them in Costa Rica, with an average of just under one child present. Slightly more than half the sample (54%) racially identified as Mestizo, 21% as white, 14% as Black, Mulato, or Afrodescendant, 4% as indigenous, and 7% as other. Half of all respondents were Nicaraguan, a third were Venezuelan, 7% Salvadoran, and the remaining 10% were from eight other countries.

The ERESS sample was also diverse with respect to migration status and histories. While 63% of participants had applied for asylum and were awaiting their asylum interview, 14% had already been granted asylum, 5% were amidst an asylum appeal process, 14% had a different (non-humanitarian) type of visa, and 4% were undocumented, including several who had been denied asylum and had received an unfavorable appeal determination. Sixteen percent of participants had been in Costa Rica for less than two years (but more than six months); 17% for 2–3 years; 37% for 3–4 years; and 30% for ≥4 years.

ERESS participants conveyed substantial socioeconomic instability since arriving to Costa Rica. By baseline, 86% had changed residences and half had been homeless in Costa Rica at least once (Table 1). Forty-nine percent reported going hungry within the week before baseline despite the fact that 79% had done something to generate income that week. Just under a quarter did “something they were not proud of to make money or survive” the week before baseline. Participants’ socioeconomic instability continued during the follow-ups. For example, in 24% and 20% of follow-ups, respectively, participants reported changing residences and being homeless. Likewise, they experienced hunger in 38% of the times we observed them even though they did something to generate income in 94% of those follow-ups. In a third of follow-ups, participants reported doing something they weren’t proud of for the sake of survival or economic necessity.

### Panel participation, attrition, and missingness

Participation in the follow-up portion of ERESS was high. Ninety-five percent of those who participated in the baseline survey completed at least one follow-up (Table 1). Modal participation (74%) continued until the last week and, on average, those who completed at least one follow-up continued to participate at least through the eleventh week (Table 1). Despite overall high participation and retention rates, it remains possible that some groups were more likely to participate, participated longer, or participated more regularly than did others. Identifying such disparities is critical to determining which groups, if any, are more challenging to track over time. To assess this possibility, we examined follow-up participation in three ways.

**Fig 4:**
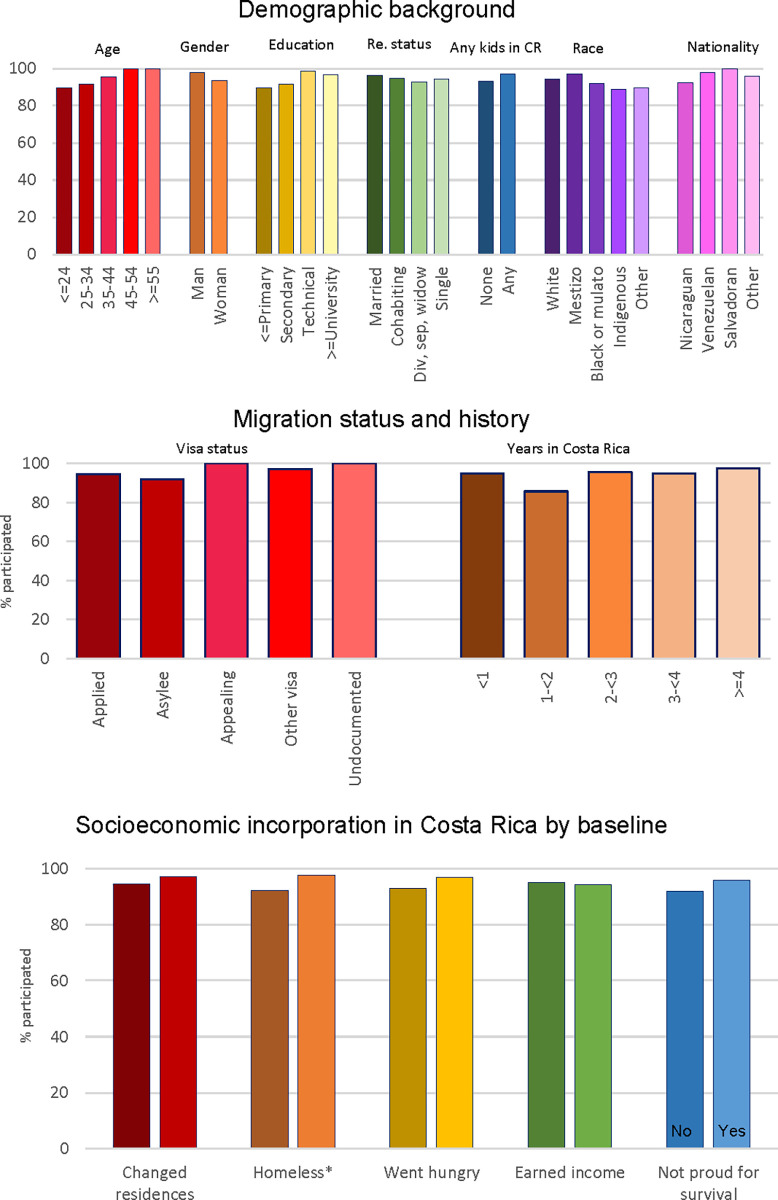
Participation rates in online follow-up surveys during. ERESS N = 260 participants. *p < .05, **p < .01, ***p < .001, two-tailed tests of significance.

First, we investigated follow-up participation rates. Specifically, we used chi-squared tests to compare the percentage of ERESS participants from different backgrounds who completed at least one follow-up survey online. The results presented in Fig 4 indicate no significant differences in participation rates along demographic lines. For example, women who completed the baseline survey were equally likely as men who completed the baseline survey to participate in follow-ups. Likewise, baseline participants with children in Costa Rica were as likely as baseline participants without children with them in Costa Rica to complete at least one follow-up survey. Comparable participation rates in the follow-up portion of ERESS were also observed across baseline participants of distinct ages, education levels, relationship statuses, races, and national origins.

Participation rates in the follow-up also did not significantly differ between baseline participants with different visa statuses or numbers of years in Costa Rica. With respect to baseline socioeconomic circumstances, we observe just one significant difference: participants who had been homeless in Costa Rica by baseline were 5 percentage points less likely to complete any online survey than were participants who had not been homeless since arriving to Costa Rica (92% versus 97%, Fig 4). Thus, with the exception of the most socioeconomically vulnerable migrants, we find similar uptake rates across a range of demographic backgrounds and circumstances.

**Fig 5:**
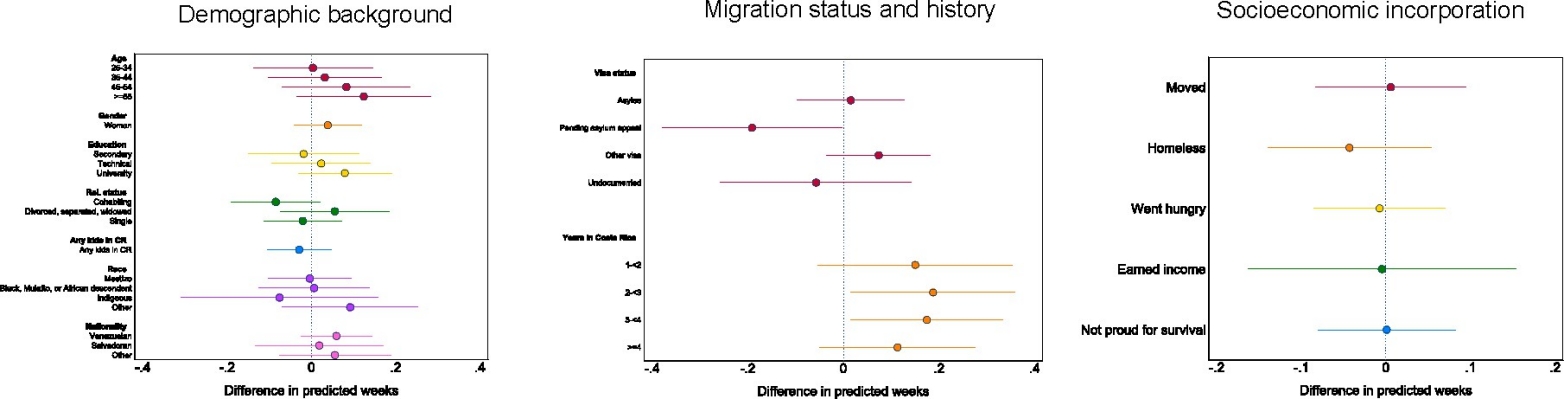
Predicted differences in duration of follow-up participation during. ERESS N = 247 ERESS participants who participated in follow-ups. *p < .05, **p < .01, ***p < .001, two-tailed tests of significance.

Second, we explored how attrition rates differed across groups by comparing the last week of follow-up participation (1 to 12) using bivariable Poisson regression. Here, we limit the analysis to individuals who completed at least one follow-up survey. As can be seen in Fig 5, we detect no significant differences in attrition rates across any demographic trait. This means that among ERESS participants who completed at least one follow-up survey online, we observe no significant differences in the timing of last follow-up (e.g. between the first and twelfth follow-up week) across gender, the presence or absence of children with them in Costa Rica, age, education, relationship status, race, or national origin.

However, we find that participants amidst an asylum appeal process stopped taking online surveys an average of .2 weeks earlier than did those who were still awaiting their asylum interview. Further, participants who had been in Costa Rica for 2 to 4 years continued to take online surveys for an average of .18 weeks longer than did those who had been in Costa Rica for less than a year by baseline. Thus, we observe slight significant differences in attrition across migration status and history. Turning to socioeconomic incorporation, we detect no significant differences in attrition between participants in the follow-up surveys who did and did not experience changes in residence, homelessness, hunger, income generation, or having to do something they were not proud of for survival at any point during their follow-ups. Taken on the whole, these results indicate that it is possible to retain MNP from diverse backgrounds at highly similar if not comparable rates and to further retain them despite ongoing economic precarity.

**Fig 6:**
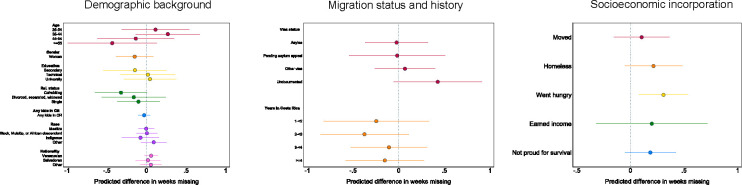
Predicted differences in number of missing weeks during. ERESS N = 247 ERESS participants who participated in follow-ups. *p < .05, **p < .01, ***p < .001, two-tailed tests of significance.

Third, we assessed differences in participants’ number of missing weeks, e.g. weeks when they did not complete the follow-up survey but had not yet completed their last follow-up. Here again we employ bivariable regression. Looking across demographic characteristics in Fig 6, we find no significant demographic differences. Put differently, among ERESS participants who completed at least one follow-up survey, we observe a comparable number of weeks when they did *not* complete follow-up surveys across gender, the presence or absence of children in Costa Rica, and all other demographic indicators. (In sensitivity analyses not shown, however, participants living with 2 or 3 children in Costa Rica respectively missed an average of .43 and .64 follow-up weeks more than participants living with no children in Costa Rica and these differences were statistically significant). No significant differences in the number of missing weeks are detected across visa status or years in Costa Rica (Fig 6). In terms of socioeconomic incorporation, we see that participants who ever went hungry during the follow-up portion of the study missed an average of .31 weeks more than those who did not. No other significant differences are detectable across any other indicator of socioeconomic incorporation. The results in Fig 6 thus echo those of our earlier analyses in that they suggest highly similar rates of follow-up completion, though slight differences are detected by participants’ experiences with hunger.

### Dynamic changes in the socioeconomic circumstances of MNP in Costa Rica

**Fig 7:**
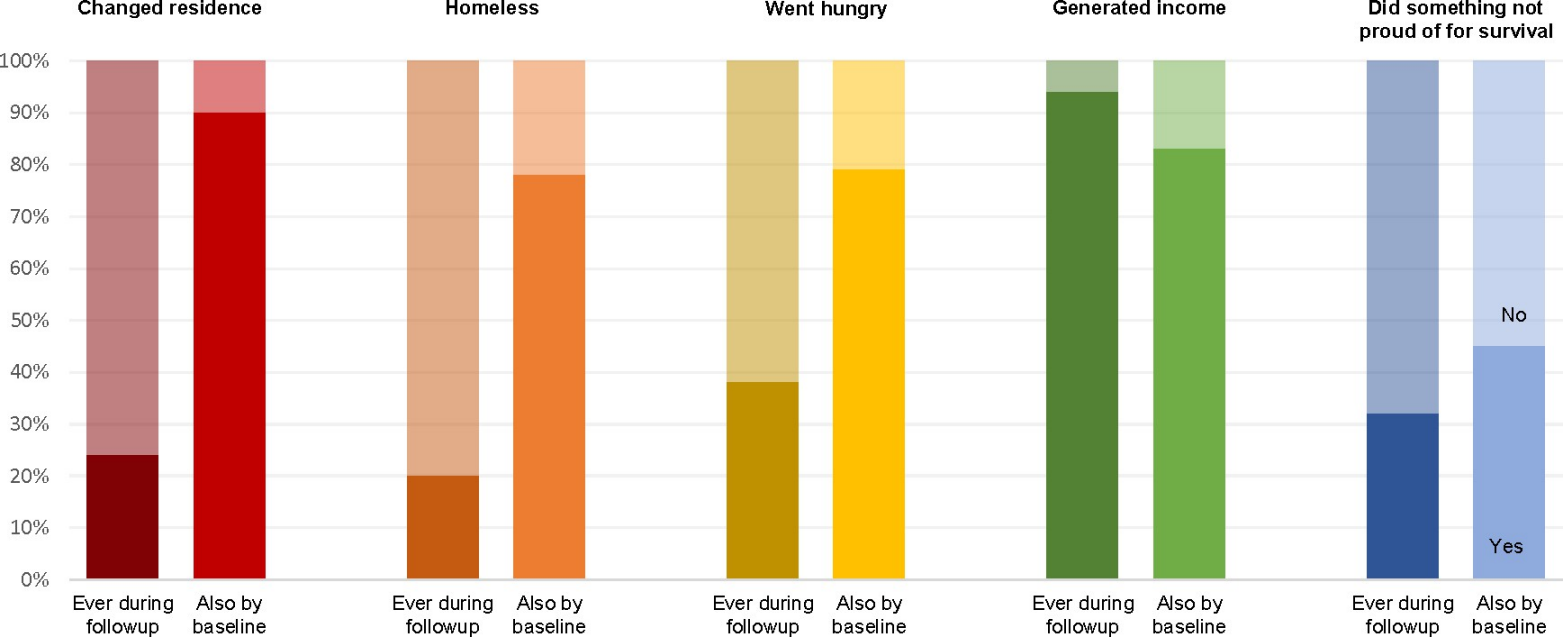
Economic incorporation during follow-up and overlap with experiences in-country by baseline. N = 247 ERESS participants who participated in follow-ups.

Having shown largely consistent participation and retention rates, we next investigate the degree of socioeconomic volatility in our sample. Descriptively, 24% of participants *ever* changed residences during the weekly follow-ups, while 20%, 38%, and 32% respectively *ever* experienced homelessness, hunger, or having to do something they weren’t proud of for survival during follow-up (darker bars shown in Fig 7). At the same time, 94% of participants *ever* generated income during the follow-up period (univariate analyses not shown).

**Fig 8:**
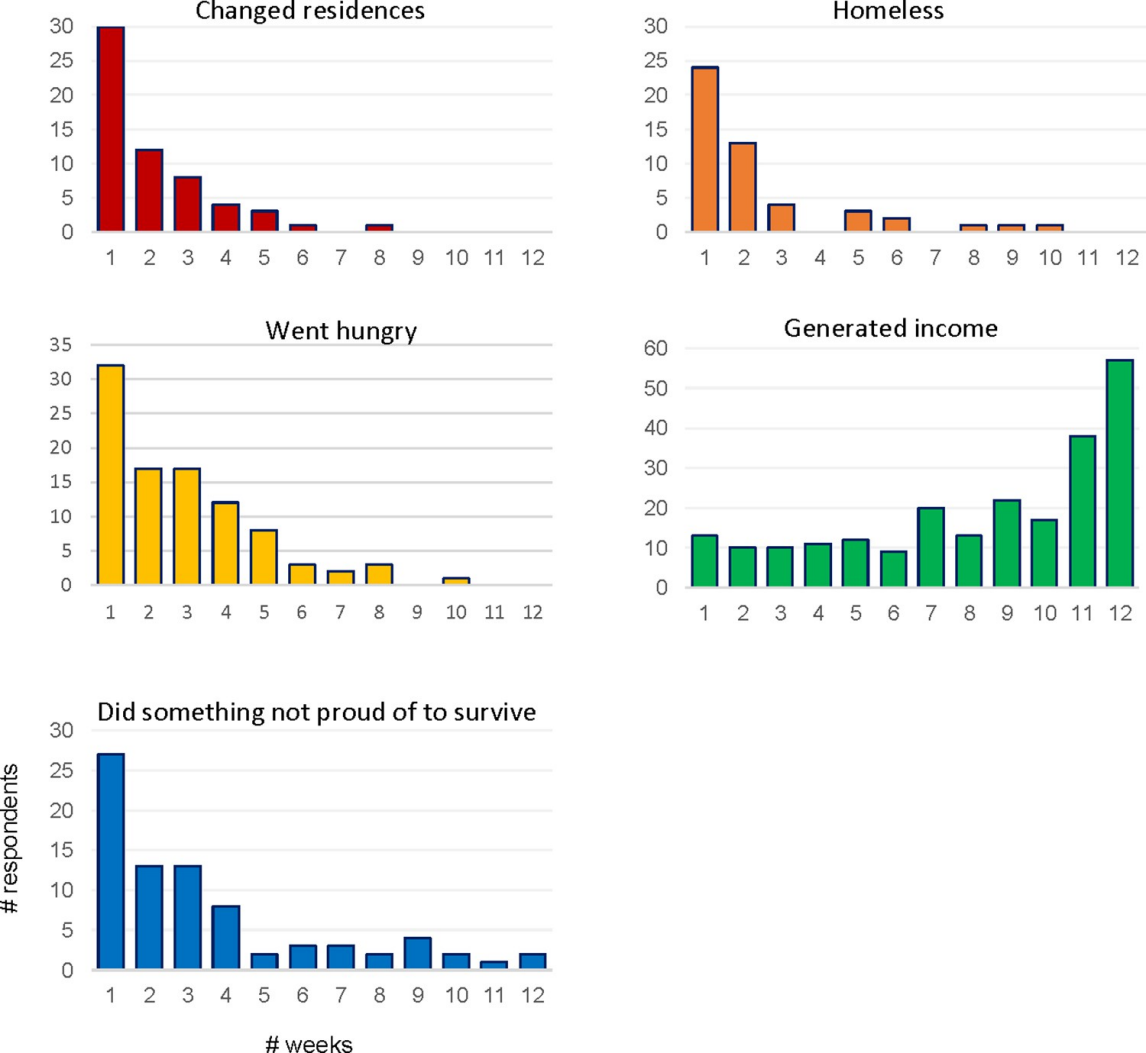
Socioeconomic volatility during ERESS follow-up. N = 247 ERESS participants who participated in follow-ups.

How frequent were these experiences? To answer that question, Fig 8 plots histograms of the number of weeks respondents reported each of these experiences and the number of weeks they reported earning an income. First, among those who ever changed residences during the follow-up period, 30% changed residences only once. The remaining 70% however, changed residences between two and eight times, suggesting a high degree of residential instability among those who ever changed residences while we observed them (Fig 8). A similar pattern emerges with respect to homelessness. Approximately 24% of participants who ever experienced homelessness during follow-up experienced it for one week only, while the remaining 76% experienced it in two to ten weeks (Fig 8). We therefore observe substantial dynamism in the housing situations of select participants in just a short window of time (~3 months). Overlap in residential instability and homelessness was only moderate: 21% of weeks participants reported changing residences they also reported homelessness; likewise, 23% of weeks they reported homelessness, they also reported changing residences.

Similar patterns are also observed in terms of hunger and doing something someone is not proud of for survival. In both cases, a minority of participants who ever had these experiences had them only once (32% and 27%, respectively), while the majority experienced them more than once but not always (Fig 8). Thus, in terms of these indicators of economic hardship, we also detect substantial temporal variability. Income generation, although a comparatively more stable experience, was nonetheless also temporally variable for many: Among those who *ever* reported generating an income, 57% generated income in 12 weeks, whereas the rest generated income less frequently.

When thinking about temporal variability, a second question worth considering is: to what extent do those reporting a given experience during follow-up also report having that same experience before baseline? Answering this question helps clarify to what extent our follow-ups capture longer-term trajectories within-participants versus their entre into first-time experiences in Costa Rica. Returning to Fig 7, the bright bars help elucidate this distinction. As can be seen from the bright red bar, 90% of participants who changed residence during follow-up had already changed residence once by baseline. This means that 10% of those who changed residences, however, experienced this change for the first time during the follow-up period. The percentages of ever-homeless and ever-hungry participants who had these experiences for the first time during follow-up were more than twice as high—22% and 21%, respectively (shown in orange and yellow, Fig 7). More than half (54%) of those who ever did something they weren’t proud of for survival had this experience for the first-time during follow-up. This latter statistic, however, should be interpreted with greater caution in light of the possibility of greater measurement error during the baseline survey, which was administered over the phone by an interviewer. Eighty-three percent of participants who generated income during follow-up were generating income at baseline, whereas the remaining 17% were not.

On the whole, the results presented in Figs 7 and 8 offer three substantive takeaways. First, a non-trivial minority of our sample experienced socioeconomic hardships during the follow-up period of ERESS. Second, most of those who reported a given socioeconomic experience during follow-up reported this experience multiple times. Finally, the follow-ups we conducted over time enabled us to capture both instances in which people *transitioned* into new experiences and when they had *repeat* experiences. This distinction is important when considering that other facets of migrants’ lives, such as their migration plans, family dynamics, and health and wellbeing, may respond differently to new versus repeated experiences.

## Conclusion

Within the Americas and across the globe, migration dynamics are rapidly changing, with refugees and other migrants in need of protection (MNP) accounting for an increasing share of all migrants every passing year. Panel micro-level data to understand the temporal dynamics of MNPs’ migration-related experience nevertheless remains glaringly sparse. A primary goal of this study was to assess the feasibility of retaining MNP from diverse backgrounds in intensive panel surveys. In an attempt to animate further data collection of this nature, we secondarily strove to demonstrate how intensive panel data like ours can address previously unanswered questions about MNPs’ incorporation, family, and health trajectories. Our approach demonstrates various advantages over existing cross-sectional data sources and methods because it allows us to capture a range of experiences that MNP face in a country of reception.

By and large, analyses of our data indicate that MNP with different demographic profiles, who have been in Costa Rica for varying amounts of time and with distinct immigration statuses, can be retained in intensive panel surveys at reasonable and comparable rates for at least twelve weeks (~3 months). They further suggest that scientists can retain MNP in surveys even when they face extreme residential and economic insecurity—situations that are likely impactful for nearly every other facet of their lives. The handful of significant differences we observe with respect to selection into, duration, and consistency of participation in longitudinal follow-ups suggests that several circumstances may, however, slightly impede participation. These include prior homelessness in-destination, which was associated with lower uptake rates of the follow-up portion of the study. They also include immigration status, as those who were appealing an asylum denial participated slightly less long than did others. Those who had been in Costa Rica for less than a year also participated for a slightly shorter duration than did others, though this difference was only significant when compared to participants who had been in-country for two to four years. Finally, ever going hungry during a follow-up survey was associated with slightly more missing weeks. Researchers wishing to collect future longitudinal data among MNP should therefore be extra attentive to participants in these situations.

These findings lend confidence in researchers’ capacity to create data sources that will engender deeper understandings of how the lives of MNP unfold abroad. At the same time, the conclusions that can be drawn from our study are limited in several ways. First, because we collected weekly panel data for a relatively short period of time—13 weeks (including baseline)—further data collection and research are needed to understand the limits of different groups’ willingness and ability to participate in intensive panel surveys beyond this duration. Ideally, if participation rates remained high for a year or more, this would enable observations of many important transitions in MNP’s lives, such as transitions in their immigration status and corresponding changes in their quality of life, family structure and household composition, as well as in their health and wellbeing. Such insights are critical to identifying, for instance, inflection points when MNPs’ residential circumstances, employment, social lives, and migration plans begin to stabilize. Likewise, they are essential to identifying the most vulnerable sub-groups who may live in perpetual instability.

Second, our sample is specifically comprised of Latin American MNP who had migrated to Costa Rica. The extent to which intensive panel data collection can work among other populations originating from and residing in different locations therefore remains an open question. Nonetheless, a growing body of qualitative research and cross-sectional data on MNP across Latin America and in Europe, Jordan, southeast Asia, and sub-Saharan Africa provides new, contextually specific insights about the opportunities and needs for intensive panel data collection with MNP originating from and living in distinct political and cultural settings [27,70–72]. Third, in order to participate in our panel survey, participants needed to access a cellphone and the Internet. Our formative focus groups indicated that MNP in Costa Rica can typically access both. Nevertheless, studies of Latin American MNP who have migrated to other countries in the region suggest that the most socioeconomically disadvantaged MNP and those living in rural areas may have a more difficult time accessing either [73–75]. It therefore remains possible that some groups of MNP are underrepresented in our study. Fourth, and relatedly, our survey results are based on a convenience sample that we recruited through a local NGO and subsequent snowballing. While our findings lack external validity, it is important to keep in mind that no reliable sampling frame for MNP exist in Costa Rica or in most countries, making it especially complicated to collect representative data.

In short, intensive panel studies like ERESS can successfully retain Latin American MNP residing in Latin American settings in a way that promises to reveal many new insights into the dynamic interplay of their experiences both pre- and post- migration. Intensive panel data collection is, however, notably complicated, costly, and time intensive. These drawbacks threaten to impede future data collection of this nature. Nevertheless, temporally and geographically expanding intensive panel data among MNP is a crucial next step to understanding how their lives unfold once abroad and to conceiving of the short and longer-term implications of rapidly shifting migration dynamics that force millions of migrants to flee their countries of origin each year.

## Supporting information

S1 Table Description of indicators. (PDF)

S2 Table Descriptive comparison of ERESS and UNHCR participants. Note: UNHCR data come from three pooled standardized surveys conducted in 2022. For more information, visit https://microdata.unhcr.org/index.php/catalog/655.
(PDF)
