# Supplementary material for: Design and implementation of an intensive panel survey with refugees and other migrants in need of protection in Costa Rica
Source: PLoS One. 2024 Mar 28;19(3):e0301135. doi: 10.1371/journal.pone.0301135 (PMC10977773; doi:10.1371/journal.pone.0301135)
Supplement: S1 Table — (PDF) [file pone.0301135.s001.pdf]

S1 Table. Description of Indicators

Variable	Survey question	Response options
Demographic background		
Age	How many years old are you?	Open-ended
Woman	What is your gender?	Male<br>Female<br>Other
Educational attainment	What is the highest education level you have passed in your life?	None<br>Incomplete primary<br>Complete primary<br>Incomplete secondary<br>Complete secondary<br>Incomplete vocational<br>Complete vocational<br>Incomplete university<br>Complete university<br>More than university
Relationship status	What is your current relationship status?	Cohabiting<br>Married<br>Partnered but not living together<br>Separated<br>Divorced<br>Widowed<br>Single
Number of children in Costa Rica	How many biological or foster children do you have?	Open-ended
	--> What are their ages?	Open-ended
	--> How many of those minor children (under the age of 18) currently live in Costa Rica?	Open-ended
Race	With which race and/or ethnicity do you identify?	Asian<br>White<br>Mestizo<br>Mulato<br>Black or Afro-descendent<br>Indigenous<br>Other (please specify)
Nationality	What country are you from?	Nicaragua<br>Venezuela<br>El Salvador<br>Honduras<br>Cuba<br>Colombia<br>Other (please specify)
Sociolegal incorporation		
Migration status	What is your current visa status? As a reminder, this survey is anonymous. This information is only for scientific purposes.	Has not applied for a visa yet<br>Applied for asylum, awaiting an interview<br>Has asylum<br>Denied asylum and is now in an appeal process<br>Denied asylum and is not in an appeal process

		Work visa<br>Tourism visa<br>Other (please specify)
Years in Costa Rica	In what year did you most recently arrive to Costa Rica without having since returned to live in your country?	Open-ended
Socioeconomic incorporation in Costa Rica		
Changed residences (by baseline/ last week)	Have you changed residences since you arrived to Costa Rica / in the last week?	Yes/ No
Homeless (by baseline/ last week)	Have you experienced a lack of housing since you arrived to Costa Rica / in the last week?	Yes/ No
Went hungry last week	Have you or someone in your family in Costa Rica had to go hungry recently (or in the last week) because they didn't have food to eat?	Yes/ No
Generated income last week	In the last week, have you done anything to earn money?	Yes/ No
Did something not proud of to survive last week	Since arriving to Costa Rica (or in the last week), have you had to do something that you were [illegible]	Yes/ No
