# Supplementary material for: Design and implementation of an intensive panel survey with refugees and other migrants in need of protection in Costa Rica
Source: PLoS One. 2024 Mar 28;19(3):e0301135. doi: 10.1371/journal.pone.0301135 (PMC10977773; doi:10.1371/journal.pone.0301135)
Supplement: S2 Table — Note: UNHCR data come from three pooled standardized surveys conducted in 2022. For more information, visit https://microdata.unhcr.org/index.php/catalog/655. (PDF) [file pone.0301135.s002.pdf]

S2 Table. Descriptive comparison of ERESS and UNHCR participants

	**ERESS** (N=260)		**UNHCR** (N=9,271)	
	Mean/ Prop.	95% CI	Mean/ Prop.	95% CI
Demographics				
Age				
<=21	.06		.07	
22-59	.89		.89	
>=60	.06		.04	
Any child dependents in Costa Rica	.50		.52	
Nationality				
Nicaraguan	.50		.76	
Venezuelan	.34		.09	
Other	.16		.15	
Gender				
Man	.33		.48	
Woman	.67		.52	
Relationship status				
Married/ cohabiting	.55		.34	
Other	.11		.04	
Single	.34		.62	
Migration status and history				
Immigration status				
Undocumented	.04		.02	
Other visa	.14		.05	
Humanitarian	.82		.93	
Year of arrival				
<=2017	.21		.16	
2018	.39		.28	
2019	.23		.24	
2020	.03		.09	
>=2021	.14		.23	
Hardships and Incorporation in Costa Rica				
Hungry	.49		.51	
Kids enrolled in school	.80		.89	
Receiving benefits from religious group	.02		.02	
Loneliness	3.10	(.49 – 5.71)	3.03	(.64 – 5.42)

Note: UNHCR data come from three pooled standardized surveys conducted in 2022. For more information, visit https://microdata.unhcr.org/index.php/catalog/655.
